# Digital health developments and drawbacks: a review and analysis of top-returned apps for bipolar disorder

**DOI:** 10.1186/s40345-020-00202-4

**Published:** 2020-12-01

**Authors:** Sarah Lagan, Abinaya Ramakrishnan, Evan Lamont, Aparna Ramakrishnan, Mark Frye, John Torous

**Affiliations:** 1 Department of Psychiatry, Beth Israel Deaconess Medical Center, Harvard Medical School, 75 Fenwood Road, Boston, MA 02446 USA; 2 Vanderbilt University, Nashville, TN USA; 3 Boston Graduate School of Psychoanalysis, Boston, MA USA; 4 Mayo Clinic, Rochester, MN USA

**Keywords:** Bipolar disorder, Smartphones, Apps

## Abstract

**Background:**

Although a growing body of literature highlights the potential benefit of smartphone-based mobile apps to aid in self-management and treatment of bipolar disorder, it is unclear whether such evidence-based apps are readily available and accessible to a user of the app store.

**Results:**

Using our systematic framework for the evaluation of mental health apps, we analyzed the accessibility, privacy, clinical foundation, features, and interoperability of the top-returned 100 apps for bipolar disorder. Only 56% of the apps mentioned bipolar disorder specifically in their title, description, or content. Only one app’s efficacy was supported in a peer-reviewed study, and 32 apps lacked privacy policies. The most common features provided were mood tracking, journaling, and psychoeducation.

**Conclusions:**

Our analysis reveals substantial limitations in the current digital environment for individuals seeking an evidence-based, clinically usable app for bipolar disorder. Although there have been academic advances in development of digital interventions for bipolar disorder, this work has yet to be translated to the publicly available app marketplace. This unmet need of digital mood management underscores the need for a comprehensive evaluation system of mental health apps, which we have endeavored to provide through our framework and accompanying database (apps.digitalpsych.org).

## Background

With the rise of digital tools and applications, smartphone apps offer promising tools to augment support and self-management for individuals with bipolar disorder (BD). With a prevalence rate of > 1% of the world’s population, patients who need chronic illness management may not have access to subspeciality clinics, and primary care providers are increasingly comfortable working with this patient population, provided supportive technology to facilitate monitoring and follow-up. (Rowland and Marwaha 2018). Studies have suggested that, as of 2019, smartphone ownership among individuals with bipolar disorder exceeds 75% (Hidalgo-Mazzei et al. 2019; Young et al. 2020), and there is evident interest in app use among individuals with BD, with 40% of young adults with bipolar disorder having used an app for symptom management and 79% of those not using an app wanting to try (Nicholas et al. 2017).

The feasibility and preliminary efficacy of mobile interventions for bipolar disorder have been validated in a variety of settings, with programs including a personal digital assistant (Depp et al. 2010), weekly text-services, and other short-message based interventions demonstrating evidence of benefit (Bopp et al. 2010). There is a robust literature base of both internet-based and smartphone-based interventions supporting self-management strategies for ongoing monitoring, education, and maintaining hope (Gliddon et al. 2017). Smartphone apps can enable both active (user-inputted) and passive (automatically collected) data collection to aid in BD diagnostics, advance evidence-based treatments like social rhythm therapy, and self-management (Torous and Powell 2015). Software applications such as Mood Rhythm and MONARCA, for example, use sensors and self-assessments in order to gain data about sleep, social activity, and mood to provide more information for both the patients and their clinicians (Matthews et al. 2016). In a 6-month, randomized, placebo-controlled, single-blind, parallel group trial utilizing MONARCA, bipolar patients who used the app, in comparison to those who did not, had fewer symptoms of mania, highlighting that both active and passive data collection may meaningfully augment conventional treatment (Faurholt-Jepsen et al. 2015). A study involving smartphone-based monitoring systems in conjunction with wrist worn accelerometers demonstrated adequate usability and feasibility (Faurholt-Jepsen et al. 2019a). Indeed, digital phenotyping—deriving metrics like location and activity patterns, social phone utilization, and symptom change—are emerging as mobile target interventions for BD (Huckvale et al. 2019). These metrics may help elucidate digital biomarkers to detect both diagnostic mood status and symptom change (Ortiz and Grof 2016), ultimately facilitating the potential for early relapse detection (Jacobson et al. 2019; Faurholt-Jepsen et al. 2019b). Survey studies have indicated that individuals with bipolar disorder are interested in and open to using apps for illness management, including apps with automatic data collection to complement traditional user-inputted metrics (Daus et al. 2018). Digital phenotyping is the latest promising avenue of exploration, adding to the robust base of literature supporting the efficacy of and receptiveness to smartphone interventions for BD.

However, these promising research findings may not translate into widely available apps that patients and clinicians can use as tools today if these digital tools are not available to an end user of the app store. While a search on the app store yields numerous results with the “bipolar” search term, it is unclear whether order in returned search is at all associated with app quality or clinical utility. Since the last systematic review of publicly available apps for BD in 2015, which highlighted serious concerns around privacy, evidence, engagement, and potential for harm, it is unknown if the landscape has meaningfully changed in response to emerging research about the potential of digital interventions for BD, and whether an average app store user is now more able to access quality, research-supported apps (Nicholas et al. 2015). Other excellent recent reviews have focused on the evidence for bipolar disorders apps based on the research literature, but what is available and being offered to patients today in the public app marketplace is likely different than the subject of this review (Bauer et al. 2020). We intend to address this lacuna in the existing literature: while recent work by our team and others has thoroughly investigated the potential of apps in research settings, far less attention has been paid to what an app store end user is able to find and access. Advances in digital health research are promising, but without widespread translation to the broader public their impact is limited. We thus sought to examine the safety, relevance, and clinical utility of the apps that are most readily available for lay person seeking tools for BD, which is also understudied when compared to technology for anxiety, depression, and other mental health conditions.

In the absence of strict oversight to help guide users find appropriate tools in the app store, we have proposed an enduring and reproducible framework to guide the evaluation and assessment of mobile health apps (Henson et al. 2019; Lagan et al. 2020). The framework is based on the American Psychiatric Association’s app evaluation model which has been well studied and utilized (Martinengo et al. 2019; Cohen et al. 2020; Bergin and Davies 2020; Ondersma and Walters 2020; Levine et al. 2020). The framework comprises 105 different questions examining app accessibility, origin, functionality, privacy, features, and clinical foundation, ultimately providing a comprehensive picture of app quality and utility. Each question in the framework corresponds to a principle in the American Psychiatric Association’s app evolution model but now is reduced to a reproducible data point or number to encourage transparency and cultural respect. Seeking to identify the attributes of the most accessible apps for bipolar disorder, we applied this framework to the 100 top-returned apps on the Apple iOS store, investigating a wide array of their features along with correspondence to evidence-based principles of BD treatment and self-management.

## Methods

Beyond appearing in the search, there was no inclusion criteria for app analysis, as an objective of this study was to assess the features of the most readily available and easily findable apps for a layperson. On February 20, 2020 the term “bipolar” was entered into the iOS app store. Of the first 107 returned apps, nine were not usable (5 required an access code; 2 were unavailable in English; 2 became unavailable over the course of the study). The remaining 98 apps were downloaded onto an iPhone 6 and iPhone 8 for complete assessment. Apps that were not free to download were purchased by raters for analysis.

Each app was assessed by at least two raters (AMR, ANR, EL, SL). Apps were evaluated based on our previously established 105 question framework created in conjunction with the American Psychiatric Association (APA). These 105 questions are based on the APA’s App Evaluation Model, with questions sorted into six categories: Origin, Functionality, and Accessibility (31 questions); Inputs and Outputs (15 questions); Privacy and Security (14 questions); Evidence and Clinical Foundation (8 questions); Features and Engagement Style (31 questions); and Interoperability and Data Sharing (6 questions). The questions are all objective and can be coded with a binary or numeric answer. The framework employed thus promotes comprehensive user testing while minimizing subjectivity and providing transparent assessment results. The ratings for each individual app are available upon request, and the apps suitable for mental health and wellness use are included in the database which can be accessed at https://apps.digitalpsych.org/Apps.

All raters underwent a 1-h training in order to complete the framework questions. Interrater reliability was assessed using Cohen’s kappa statistic (McHugh 2012). Following the training, the majority of raters demonstrated very good interrater reliability (defined as a kappa value of above 0.75), with an average kappa of 0.84 across the first five apps rated. Discrepancies between the two raters were initially addressed one-by-one in discussion and used to clarify the description of each question, and subsequently rectified by a second look at the source of the discrepancy (either app store information, privacy policy, or in-app features and functionality). All training materials have now been published online alongside the database, enabling any interested user to undergo the training and become an app rater. The resulting data was analyzed with descriptive statistics.

## Results

**Fig. 1:**
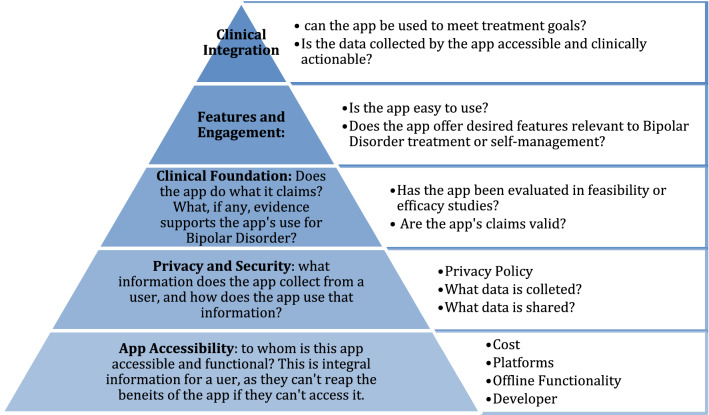
Framework levels and illustrative questions. The pyramid depicts the major considerations at each level of the framework and includes illustrative questions or themes at each level

Of the first 107 bipolar disorder related apps, nine (8%) were inaccessible without an access code or unavailable in English, and thus, a total of 98 iOS bipolar disorder apps were evaluated with our framework (Fig. 1).

### Function and relevance

In the iOS Store, apps returned on a search bipolar disorder (BD) were categorized in Business (n = 1), Education (n = 4), Entertainment (n = 2), Games (n = 13), Health and Fitness (n = 34), Lifestyle (n = 19), Magazine and Newspaper (n = 1), Medical (n = 19), Productivity (n = 2), Social Networking (n = 3), and Stickers (n = 2). The range of primary app functions reflects that the top returned apps are not necessarily patient facing or relevant to an individual with bipolar disorder. Only 56 apps (57%) explicitly mentioned bipolar disorder in their app description, title, or content.

### Origin and accessibility

The framework’s questions around origin and accessibility offer a comprehensive picture of who can download and access the app, including considerations like availability across different platforms, cost, offline functionality, last update, app size, and availability in different languages.

Of the 98 apps top returned iOS apps, 35 apps were also available on the Google Play store. 72 apps were free to download, although 41 of these apps required an in-app purchase or required a subscription to access the full swath of content. Of the 22 apps that were not free to download, the minimum cost was $0.99 USD and the maximum cost was $49.99 USD with a median cost of $1.99. Sixty six apps did not require an active internet connection after download and could be accessed offline, while 32 apps required internet connection after download to access content.

Many of the apps were infrequently downloaded and not currently updated, prompting concern given that lack of update in the last 180 days—a metric associated with lower app quality (Wisniewski et al. 2019a). Only 38 apps had been updated in the last 180 days in the iOS Store, with 10 apps still in the first version. The average rating for analyzed apps was 4.2 in the iOS store and 4.0 in the Google Play store. 44 apps had over 20 reviews in the iOS store and 23 apps had over 20 reviews in the Google Play store. The fact that more than half of the apps available on iOS had fewer than 20 reviews suggests that apps returned on search for BD may not be widely downloaded (the iOS app store does not provide direct data on number of downloads). The average app size was 47.5 MB in the Apple Store and 20.6 MB in the Google Play store, and 20 apps were available in at least one other language in addition to English, with the majority (n = 19) of these offering Spanish functionality.

Regarding app origin, only two apps were affiliated with a university or healthcare organization, and both were treatment guideline apps intended for physicians. None of the top 100 apps had government affiliation, although several governmental organizations, including the Department of Veterans Affairs and Department of Defense, have ventured into the mental health app space and developed apps (Owen et al. 2018). None of the apps that had been assessed in research studies for effectiveness at BD management or treatment appeared in the top 100.

### Inputs and outputs

Questions about inputs and outputs help illustrate the kind of data that each app collects and returns to the user. The most common inputs among the 98 apps analyzed were surveys (n = 48), diaries or user inputted text entries (n = 38), geolocation (n = 9), and camera (n = 9). Six of the apps that collected geolocation data from a user’s phone were mood-tracking apps, while two were apps with a peer support or community forum. The most common outputs were notifications (n = 49), summaries of data (n = 41), graphs of data (n = 40). 8 apps provided a link to formal care or coaching within the app itself.

### Privacy and confidentiality

Privacy policies were available to the public in 66 of the 98 apps, through either a link from the app store description or within the app itself. 27 apps (40.9%) of apps with privacy policies mentioned the disclosure of users’ personal information to third parties. Although several apps included features that prompted users to enter personal health information such as medication tracking (n = 5) alongside identifying data, only one app claimed to be HIPAA compliant.

### Clinical foundation

Questions about clinical foundation assess each app’s veracity of claims, support in peer-reviewed studies, and potential to cause harm. 93 apps in our analysis provided what they claimed, while 5 apps did not meet their claims, failing to offer the features that were advertised in their app description. One app’s description read, for example, “stimulate vital areas of the brain and heal naturally…prepare yourself for Coronavirus!” despite providing no psychoeducation or links to information about COVID-19 (Brainwaves Psychological 2020). Another claimed to provide meditation and yoga, but instead primarily served as a game without offering comprehensive meditation modules.

Only one app had supporting feasibility and efficacy studies, with 7 Cups of Tea backed by one feasibility study (Baumel and Schueller 2016) and two efficacy studies (Baumel et al. 2018; Baumel 2015), none of which involved participants with bipolar disorder specifically. Although another app, Daylio, was the subject of an article in JMIR’s mHealth (Chaudhry 2016), the article offered an in depth description of the app without a supporting feasibility or efficacy study and thus was not included in our analysis.

12 apps were rated as capable of causing harm to a user. 11 of these potentially harmful apps offered unmoderated forums or information that was triggering and not aligned with current treatment guidelines. One app, for example, encouraged users to “break the silence on your hopelessness and depression by speaking up in a small way and using a background that reflects your inner misery and despair” and provided phone wallpapers with messages including, “I disappoint myself” and “I don’t like what I’m becoming” (Wallpapers 2020). The other app was a “bipolar test and personality quiz” that offered users a “test” for bipolar disorder (Bipolar Test 2020). The questions, however, did not align with the clinically-validated Mood Disorder Questionnaire (MDQ) and even if a user obtained a result confirming they were at high risk of BD, the app provided no links or references and did not direct users to a medical professional or other resources.

### Features and engagement style

**Fig. 2:**
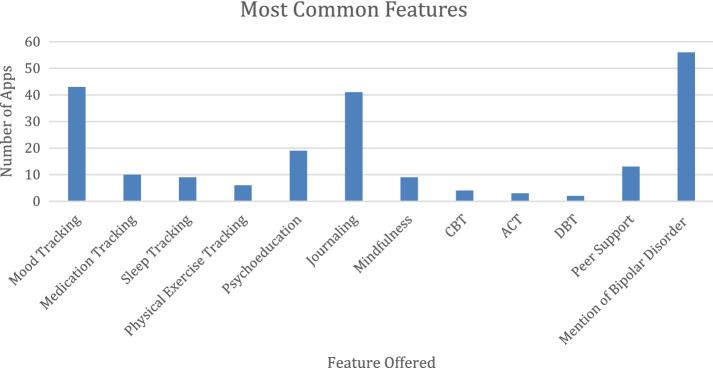
Most common features among returned apps. This figure depicts the most common features offered by apps that appeared in search for bipolar disorder

Mood-tracking, journaling, and psychoeducation were the most common features offered by apps returned in the search for bipolar disorder. 43 apps offered mood-tracking, 41 apps provided a platform for journaling, and 19 apps provided psychoeducation about either BD specifically or coping strategies and treatments more broadly. The most common engagement style was through user-generated data (n = 44), gamification (n = 23), and peer support (n = 13). Features varied significantly as a function of cost (*χ*^2^ = 15.982, p = 0.042), with apps that were totally free more likely to offer screeners or assessments, and apps with either up-front costs or in-app purchases more likely to provide meditation and mindfulness (Table 1, Fig. 2).

**Table 1:** Common app features and functionalities

App feature	Number of evaluated apps with feature (%)
Mood tracking	43 (43.9%)
Medication tracking	10 (10.2%)
Sleep tracking	9 (9.2%)
Physical exercise tracking	6 (6.1%)
Psychoeducation	19 (19.4%)
Journaling	41 (41.8%)
Mindfulness	9 (9.2%)
CBT	4 (4%)
ACT	3 (3%)
DBT	2 (2%)
Peer support	13 (13.3%)
Mention of bipolar disorder	56 (57.1%)

## Discussion

Our analysis reveals that a simple app store search may not be sufficient for an individual seeking to find an app suitable to BD education, management, or treatment, as many apps in the top 100—including paid apps—were irrelevant or raised concerns that warrant a cautious approach to app selection.

Some apps offered harmful or misleading content. The order that the search returned apps was not indicative of clinical utility, as some misleading, stigmatizing, and dangerous apps appeared before apps with features suitable to BD management and treatment. One app encouraged users to “break the silence on your hopelessness and depression by speaking up in a small way and using a background that reflects your inner misery and despair” and provided phone wallpapers with messages including, “I disappoint myself” and “I don’t like what I’m becoming” (Wallpapers 2020). Another app’s description read, “stimulate vital areas of the brain and heal naturally…prepare yourself for Coronavirus!” despite providing no psychoeducation or links to information about COVID-19 (Brainwaves Psychological 2020). One app offered no features besides downloadable stickers of an “unpredictable bipolar bear” depicted in cartoon imagery (The Bipolar Bear Bonacorso 2020).

Only one app that appeared in the search had supporting feasibility and efficacy studies, and even those peer-reviewed publications did not involve individuals with Bipolar Disorder, instead focusing on the app’s ability to mitigate symptoms of depression. While the overtly incorrect claims by some apps are a serious area of concern, even subtler claims made by commercial apps, such as “manage your symptoms of Bipolar Disorder!” should be approached cautiously given the lack of evidence across all returned apps.

Another challenge was that some apps offered what appeared to be evidence-based interventions, but upon closer inspection were likely not. For example, thirteen apps claimed to offer peer support in some form; nine of them, however, did so via unmoderated forums, where users were able to post and view content posted on a public forum, or the “moderators” did not intervene until after a comment was reported. One mood tracking app, for example, automatically published all mood and diary entries to a public newsfeed and required an in-app purchase in order to access a private diary that would not be published (Moodtrack Social Diary 2020). In all of these apps, the risk of triggering, non clinically-useful content was concerning. The concept of peer support was also defined loosely. While certified peer specialists have been shown to improve treatment outcomes across a range of mental health conditions (Felton et al. 1995), no apps we reviewed utilized certified peer specialists, instead defining “peer” to be anyone else using the app. Integrating peer specialist support into technology is a continuing area of research, with preliminary evidence of both feasibility and efficacy, but our analysis reveals that these advances in peer specialist technology research have yet to be translated to the area of publicly available BD apps (Fortuna et al. 2018).

The lack of privacy policies and specifically and the lack of HIPAA compliant apps further underscores the necessity of a cautious approach to app selection. Previous literature has highlighted the numerous risks around data disclosure of behavioral data by mental health apps (Bauer et al. 2017). Our study of 98 BD apps found that 32.7% of apps did not have a privacy policy readily available to users either through the App Store or in the app itself. Moreover, of the apps with a privacy policy, the average reading grade level was 12.1 (SD 2.5), with only 7 apps having a grade level of 9th grade or lower and 34 apps having a collegiate reading grade level or higher. While privacy and security remain important features to users (Dehling et al. 2015), the lack of transparent policies that require college-level literacy indicate the need to improve the state of privacy and transparency among BD apps.

### Comparison with prior work

This study builds upon numerous prior efforts in the area of mental health apps, allowing an analysis of potential changes in the space. Compared to the review of BD apps in 2015 by Nicholas et al., we employed fewer search terms, utilizing only “bipolar” instead of “bipolar,” “manic depression”, “mood swings”, and “mood.” By using this singular search term, our objective was to assess the apps that would be most readily accessible for an individual searching for a BD app. When including the top 98 returned apps in our analysis, we found that 43% of apps did not even mention Bipolar Disorder in their app title, description, or content. Like Nicholas et al., we found symptom monitoring tools such as mood-tracking and journaling to be most common among reviewed apps; in contrast to the 2015, review, however, we identified 13 apps providing community or peer support—a significant increase from the 4 such apps five years ago. Another major improvement is around privacy policies. A striking finding from Nicholas et al. was that only 18 or 82 apps had a privacy policy—a figure that has noticeably advanced, with 66 of the 98 apps we reviewed now possessing a privacy policy. Our team has done smaller app evaluation studies were we looked at the top 10 apps for bipolar disorder as returned by an app store-search (and other conditions), but results here are different as they seek to quantify the state of the field beyond the highlights of the app stores (Wisniewski et al. 2019b; Mercurio et al. 2020).

In terms of evidence-based apps, however, the commercial app space has not significantly progressed: as in the 2015 review by Nicholas et al., we identified just one app supported by feasibility or efficacy studies. This finding suggests that research around digital tools for Bipolar Disorder has not been translated into many evidence-based, clinically relevant apps on the app store. As the need for digital resources becomes increasingly urgent in the wake of the COVID-19 pandemic, the app store content most available to end users is of the utmost importance.

Overall, while our analysis demonstrates that some strides have been made in the landscape of apps for bipolar disorder since 2015, a noticeable gap between research and practice is still present. Despite the body of research highlighting the potential of apps to support individuals with bipolar disorder, such research has yet to be translated to the publicly available app marketplace, where only one app that appears in a user’s search for “bipolar” is backed by supporting studies. Although there is evidence that individuals with bipolar disorder are interested in apps with automatic data collection to aid in symptom management, few of the apps utilized passive data (Daus et al. 2018). And while the effectiveness of peer support has received growing attention, available “peer support” apps have yet to progress beyond a potentially triggering community forum model. If the potential of technology is to be fully harnessed, research-backed apps must be made available to the public, and a comprehensive app evaluation system is urgent in light of limited regulation from the app stores.

### Limitations

This study employed a single search term, “bipolar,” as we sought to analyze the most immediately accessible apps for a layperson seeking BD resources in the app store. Utilizing this limited search term, however, prevented a full perusal of apps that may be relevant for an individual with bipolar disorder; it is possible that mood-tracking, mindfulness, and other tools for self-management may not explicitly reference BD at all but nonetheless offer clinical benefit. For example, the app HealthRhythms was designed to target bipolar disorder but does not return in any search for the term (Measure Health 2020). Recognizing the limited scope of this work, we view our analysis as a marker of the current state of the field and call for better processes in finding a relevant, clinically usable app via the app store for an individual seeking resource for Bipolar Disorder. This review is just one component of our broader effort to link clinicians and patients with safe, effective apps. Our database of mental health apps enables users to filter and find apps based on desired characteristics and thus connects individuals to tailored tools more effectively than a simple app store search (Nicholas et al. 2015).

Additional limitations arise from individual differences in the algorithm that determines which apps appear in what order on an iOS store search. The algorithm in fact changes daily, as a search a week later conducted on the same phone yielded the same apps but in a slightly different order. A growing body of literature highlights the rampant turnover characterizing the app space, with, for example, a clinically relevant app for depression becoming available every 2.9 days (Larsen et al. 2016). Given the dynamic nature of the app store, and the increasing focus on developing technology to support mental health (Monteith et al. 2016), it is possible that clinically relevant apps for BD have emerged in between drafting and publication of this study.

## Conclusion

Despite both the continued proliferation of mental health apps and promising research around the efficacy of smartphone apps for management and treatment of bipolar disorder, our study highlights how it remains difficult for an individual seeking a relevant app for BD to find an appropriate tool in the app store. The primary shortcoming is that users must wade through irrelevant, misleading, and even potentially dangerous apps to find a relevant one. The lack of privacy protection and transparency around user data, along with the lack of supporting evidence among available apps and potential for misleading content, all raise concerns about the most accessible public facing apps and highlight the need for a way to evaluate apps beyond app store metrics.

We employed a framework for app assessment that is research based and entirely reproducible, paving the way for future analyses of health apps and providing a tool to help clinicians, patients, and the wider public reap the benefits of digital health. All of our results are available to the public on our database that is informed by our evaluation model. Recognizing the limitations of this study, we regularly update our database to reflect the changing nature of available apps and emergence of new ones. We encourage crowd-sourcing and collaboration around app evaluation in order to provide clarity amidst the profusion of available apps for end users, ultimately equipping them to make an informed choice around an app to help them meet their goals.

## Data Availability

The full dataset generated and analyzed during the current study is available from the corresponding author upon request. App evaluations are also published on our database of mental health apps (https://apps.digitalpsych.org).
